# Lower level of complement component C3 and C3a in the plasma means poor outcome in the patients with hepatitis B virus related acute-on-chronic liver failure

**DOI:** 10.1186/s12876-020-01258-3

**Published:** 2020-04-15

**Authors:** Qian Li, Qing Lu, Meng-Qi Zhu, Chong Huang, Kang-Kang Yu, Yu-Xian Huang, Xu Zhao, Xing-Guang Luo, Jian-Ming Zheng

**Affiliations:** 1 Department of Infectious Diseases, Huashan Hospital, Fudan University, No.12 Wurumqi Middle Road, Jing’an district, Room 508, Shanghai, 200040 People’s Republic of China; 2 Institute of Antibiotics, Huashan Hospital, Fudan University, Shanghai, 200040 China; 3 Institute for Biomedical Sciences, Georgia State University, Atlanta, GA USA; 4 Department of Genetics, Yale University School of Medicine, 333 Cedar Street, New Haven, CT 06510 USA

**Keywords:** Complement, Hepatitis B virus, Acute-on-chronic liver failure

## Abstract

**Background:**

The purpose of this study is to investigate whether or not the complement system is systemically activated and to specify the clinical and prognostic implications of its components during hepatitis B virus related acute-on-chronic liver failure (HBV-ACLF).

**Methods:**

Blood samples were taken from twenty-seven patients diagnosed with HBV-ACLF, twenty-five patients diagnosed with chronic hepatitis B but without liver failure (CHB), and nine healthy volunteers (the control group). Plasma complement components were measured with Enzyme-linked immunosorbent assay. Correlative analysis were assessed between the levels of complement components and the liver failure related index.

**Results:**

The concentrations of C3 was 6568 μg/ml in the HBV-ACLF group, 8916 μg/ml in the CHB group and 15,653 μg/ml in the control group, respectively (*P* <  0.05). The concentrations of C3a was 852 ng/ml in the HBV-ACLF group, 1008 ng/ml in the CHB group and 1755 ng/ml in the control group, respectively (*P* <  0.05). The concentrations of C1q was 50,509 ng/ml in the HBV-ACLF group, 114,640 ng/ml in the CHB group and 177,001 ng/ml in the control group, respectively (*P* <  0.05). The concentrations of C1q, C3, C3a, C4, C4a and sC5b-9 were significantly higher in the control group than those in the HBV-ACLF group (3.5, 2.4, 2.1, 1.4, 1.3 and 6.0 fold, respectively). However, there was no statistical significance of the differences in the plasma concentrations of mannose binding lectin and factor B between the HBV-ACLF group and control group. The levels of C3 and C3a were inversely correlated with MELDs or CLIF-C OFs (*P* <  0.05).

**Conclusions:**

Our analysis demonstrated that the activation of the classical pathway mediated by C1q may play an important role in the pathogenesis of HBV-ACLF. Furthermore, the plasma levels of C3 and C3a may be potential novel biomarkers in predicting the outcome of HBV-ACLF.

## Background

The acute-on-chronic liver failure (ACLF) is characterized by hepatic failure and/or by multi-organ failure accompanied by high 28-day mortality. ACLF is a serious disease that many patients need liver transplantation as an option for treatment. Hepatitis B virus (HBV) is still the main reason for ACLF in the Asian region [1].

The pathophysiology of the ACLF had not been fully explored before. Several reports have observed that systemic inflammation is a hallmark of ACLF [2]. ACLF patients showed higher plasma levels of pro-inflammatory cytokines [such as the interleukin- (IL-) 6 and tumor necrosis factor- (TNF-) α], white blood cell count and C reactive protein (CRP) than in healthy individuals [3, 4]. The inflammatory inducers could be both exogenous and endogenous factors, including the microbial and nonmicrobial factors, such as the context of the tissue damage or injury. The complement system plays an important role in both host innate immune defense and inflammatory progress of human diseases [5–10]. The activation of the complement system in response to invading pathogens is initiated through the classical, the alternative and the lectin pathways. The initiators of the three activation pathway are C1q, Factor B (FB) and Mannose binding lectin (MBL), respectively. The complement activation results in the C3 cleavage, the anaphylatoxins C3a and C5a release, and the formation of a membrane attack complex (sC5b-9) to lyse the target cells. The liver is the major site for complement synthesis [11]. The complement system intensified the susceptibility of steatotic livers to ischemia and reperfusion injury. The complement component 3 (C3) deficiency, the inhibition of complement receptor 2-complement receptor 1-related protein y (an inhibitor of C3 activation) could provide the protection from hepatic ischemia and reperfusion injury in mice [12, 13]. In addition, the activation of the complement system plays a key role in hepatic inflammation and progression of injury during the pathogenesis of acetaminophen-induced hepatotoxicity [14]. However, whether or not the complement system plays the pivotal role in the pathogenesis of HBV-related ACLF (HBV-ACLF) is still unknown [15]. To our knowledge, only one study reported serum levels of the complement 3 and 4 (C3 and C4), and the complement function (CH50) in HBV-ACLF patients and the result suggested C3 level can be a prognostic marker for the mortality of the disease [15].

In nonalcoholic fatty liver disease, there is a widespread activation of the complement system which is related to the disease severity [16]. Whether the complement system has a relationship with the prognosis in HBV-ACLF or not remains largely unknown. The model for end-stage liver disease (MELD) is a conventional scoring system used as a prognostic tool devised for the end-stage liver disease and the utility of transplantation [17]. However, our previous study revealed that chronic liver failure (CLIF) consortium organ failure score (CLIF-C OF) enables more accurate prediction of short-term mortality in patients with HBV-ACLF than MELD, CLIF sequential organ failure assessment score (CLIF SOFA), and CLIF consortium ACLF score (CLIF-C ACLF) [18]. Therefore, the correlative analysis between the complement components levels and the liver failure related index, including prognostic scoring system, such as MELD and CLIF-C OF, were assessed in this study.

The purpose of this study is to investigate whether or not the complement system is systemically activated as well as its relationship with the prognosis in HBV-ACLF. The levels of the complement components plasma samples from HBV-ACLF patients were determined and compared with those in the control group. Correlative analyses between the complement components levels and the liver failure related index were also examined.

## Methods

### Patients selection, clinical sample collection and processing

We tested 27 consecutive samples from HBV-ACLF patients and 25 samples with chronic hepatitis B patients. They were treated at the Department of Infectious Diseases, Huashan Hospital, Fudan University (Shanghai, China) from February 2014 to June 2017. Exclusion criteria were: 1) patients aged less than 14 years old; 2) patients co-infected with human immunodeficiency virus; 3) patients with the coexistence of liver injury caused by any other etiologies including hepatitis C or D virus infection, drug intake, alcohol consumption and autoimmune hepatitis, etc. and 4) pregnancy and lactation (Table 1).

**Table 1:** Baseline characteristics of the HBV-ACLF, CHB group and health control groups

Characteristics	Control group	CHB group	ACLF group	*P* Value
Patients, no.	9	25	27	
Age, y (median, range)	37 (29–50)	31 (15–55)	42 (24–59)	0.1182
Gender, M/F	5/4	16/9	23/4	0.1510
ALT (IU/L) (median, range)	19 (15–38)	25 (13–106)	230 (17–2175)	< 0.0001
Bilirubin (μmol/L) (median, range)	10.5 (8.3–23.3)	12.2 (5.2–27.8)	314.9 (20–830.1)	< 0.0001
Albumin (g/L) (median, range)	ND	46 (33–49)	32 (27–42)	< 0.0001*
Creatinine (μmol/L) (median, range)	78 (47–93)	73 (36–80)	67 (21–646)	0.8256
INR	ND	1.03 (0.90–1.10)	1.90 (1.07–4.93)	< 0.0001*
AFP (ng/ml) (median, range)	ND	2.02 (1.07–348.2)	59.98 (3.85–959.8)	< 0.0001*
HBeAg positive, n(%)	0	13 (52%)	17 (63%)	0.9506*
HBV DNA (log_10_ IU/ml) (median, range)	ND	7.453 (2.699–8.656)	4.189 (2.699–7.477)	0.0178*
MELDs (median, range)	ND	2 (2–6)	23 (10–43)	0.0005*
CLIF-C OFs (median, range)	ND	6 (6–6)	8 (6–12)	Cannot be calculated

Continuous variables were presented as median and the range was shown in brackets. ND: not detected. * If the test was not detected, the other two group was compared

The diagnosis of ACLF was based on the criteria formalized by the acute-on-chronic liver failure consensus recommendations of the Asian Pacific Association for the Study of the Liver (APASL) 2014 [1]. Acute liver failure is generally defined as the development of hepatic encephalopathy within 4 weeks of onset of jaundice [1]. Since the basic premise in ACLF is to identify patients with chronic liver disease or cirrhosis presenting as acute liver failure, the time frame for liver failure was kept as 4 weeks. Acute-on-chronic liver failure is defined as the coagulation abnormality usually with an international normalized ratio (INR) ≥1.5 and total bilirubin≥10 mg/dl in this study. The diagnosis of chronic hepatitis B (CHB) was defined as the presence of HBsAg in serum for more than 6 months, high levels of HBV DNA, and by persistently elevated serum alanine aminotransferase without the other reasons [19]. The patients in chronic hepatitis B group were without liver failure. The control group was made up of nine healthcare volunteers. Venous blood was collected from the patients during first day after admission, then centrifuged, with single use aliquots of these samples were stored at − 80 °C. The study was performed in accordance with the 1964 Declaration of Helsinki and was approved by the Ethical Committee of Huashan Hospital, Fudan University. If the participants were less than 18 years old, the written consent was instead obtained from the parents/legal guardians of these participants on their behalf.

### Clinical characteristics and biochemical parameters

Clinical characteristics including all the components of various prognostic scores and blood parameters (routine blood tests, coagulation function tests, serum electrolyte levels, liver and renal function tests, and arterial blood gas analysis) were analyzed.

### Prognostic scores

The CLIF-OF score has been shown to accurately predict short-term mortality in patients with HBV-ACLF in our previous study [18]. We used the published formula to compute CLIF-C OFs and MELDs in our cohort [2, 17, 20]. The CLIF-C OFs (range 6–18) at diagnosis was defined by the presence of hepatic, renal, cerebral, coagulatory, circulatory and respiratory failure [20]. MELD score was calculated as following: 9.6 × ln [creatinine (mg/dL)] + 3.8 × ln [bilirubin (mg/dL)] + 11.2× ln (INR) + 6.4 × (etiology: 0 if cholestatic or alcoholic, 1 otherwise) [17].

### Elisa

Human Complement C1q, FB, MBL, C3, C3a, C4, C4a and sC5b-9 were measured with commercial ELISA kits (Cusabio Biotech, Wuhan, P.R. China; BD biosciences, San Diego, USA; Abcam, Cambridge, UK, QuidelSan Diego, USA; R&D Systems, Minneapolis, USA) [7]. Experiment protocols were followed according to the manufacturer’s instructions.

### Western blot

For comparison of C3 activation in plasma samples, about 2 μl of plasma samples were loaded on an SDS-PAGE gel. Samples were transferred to polyvinylidene difluoride membranes. The membranes were blocked and then incubated with mouse anti-human C3/C3b/iC3b/C3dg monoclonal antibody (1:1000, Cedarlane, Ontario, Canada) in TBST (Tris-buffered saline supplemented with Tween 20). Blots were then washed and incubated with goat anti-mouse IgG (1:15000, LI-COR Bioscience, Nebraska, USA) diluted in TBST. The membranes were developed with an Odyssey system (Li-COR Bioscience) according to the manufacturer’s protocol.

### Statistical analysis

Statistical analyses were performed with the Graphpad 5.0 (Graphpad Software, San Diego, CA, USA). Variables were expressed as mean ± standard deviation unless otherwise specified. Differences in the parameters were compared by using the non-parametric Mann-Whitney U-test and the relationship between two variables was assessed by Pearson correlation or Spearman’s rank correlation, respectively. A two-tailed *P* value of < 0.05 was considered statistically significant. Survival probabilities were estimated by means of Kaplan-Meier’s method and were compared by the log-rank test. The performance of C3 and C3a/C3 on the prediction of 28-day mortality was assessed by the receiver operating characteristic (ROC) curve.

## Results

### Baseline characteristics

All patients were *Han Chinese*. Table 1 shows the baseline characteristics at enrollment in the study. There were no significant differences in gender, age and creatinine in all groups. Nevertheless, the levels of alanine aminotransferase (ALT), total bilirubin, INR, AFP and MELD scores were significantly higher in ACLF group than that in the other groups. Unfortunately, five patients with HBV-ACLF were dead. Two patients with HBV-ACLF received liver transplant finally. Thus, seven of 27 (25.9%) patients were defined as ACLF non-survival group. Twenty patients with HBV-ACLF were survived without liver transplant, were defined as ACLF survival group. Fifteen patients with HBV-ACLF developed a variety of infections, including 6 cases of pulmonary infection.

### Complement components in plasma samples

The plasma concentrations of C1q were markedly reduced in HBV-ACLF than those in CHB group or in control group, but not the MBL and FB.

**Fig. 1:**
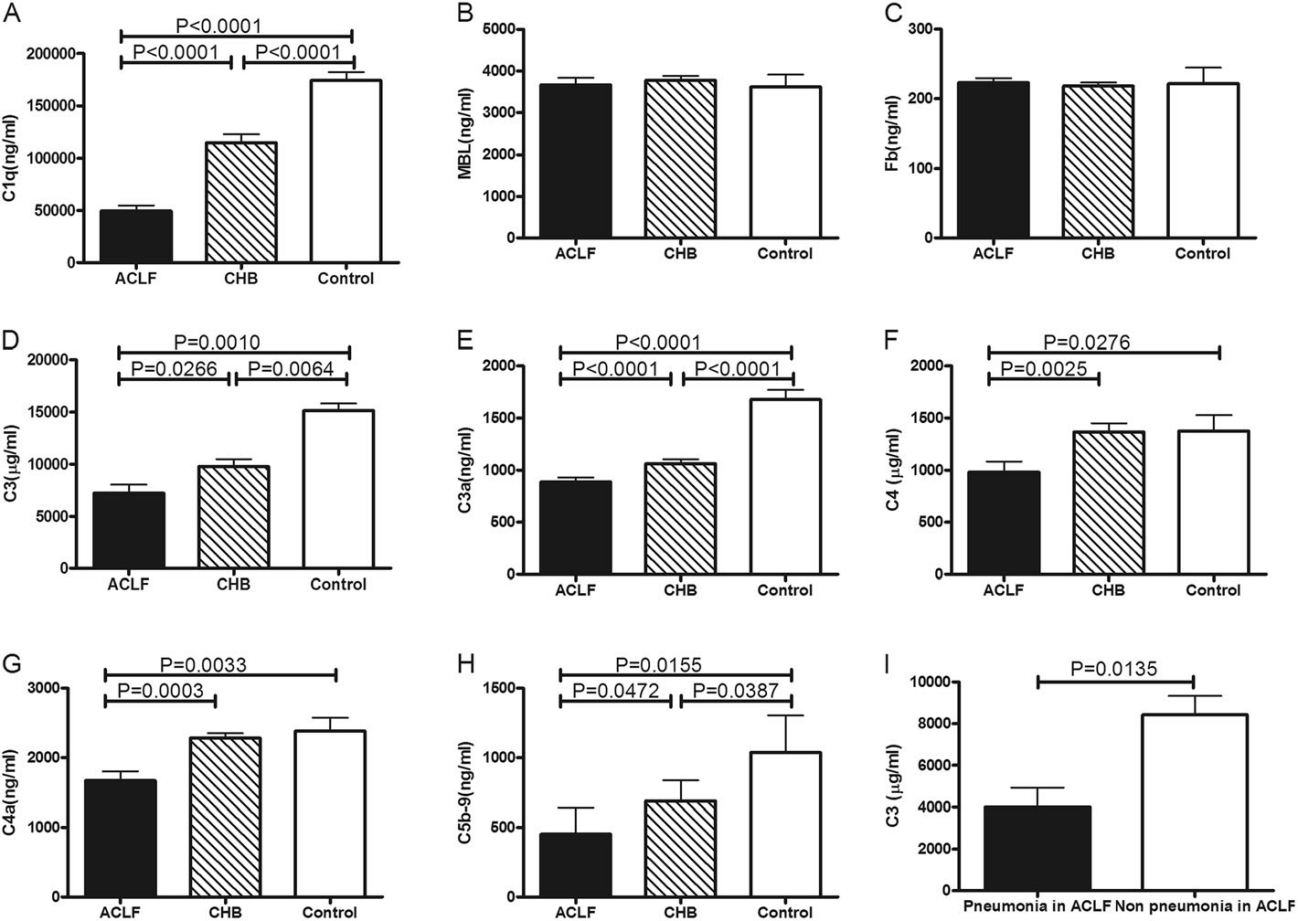
Complement in HBV-ACLF group, CHB group and control group. **a** The concentrations of C1q were significantly lower in HBV-ACLF group than that in CHB group or in control group (*P* < 0.05). **b** There was no statistical significance of the plasma concentrations of MBL in all groups. **c** There was no statistical significance of the plasma concentrations of FB in all groups. **d** Complement C3 was significantly higher in the control group than that in the HBV-ACLF group (*P* < 0.05). **e** Complement C3a was significantly higher in the control group than that in the HBV-ACLF group (*P* < 0.05). **f** Complement C4 was significantly higher in the control group than that in the HBV-ACLF group (*P* < 0.05). **g** Complement C4a was significantly higher in the control group than that in the HBV-ACLF group (*P* < 0.05). **h** Complement C5b-9 was significantly higher in the control group than that in the HBV-ACLF group (*P* < 0.05). **i** The concentrations of C3 were significantly lower in the HBV-ACLF patients with pulmonary infection than that in the HBV-ACLF patients without pulmonary infection (*P* < 0.05)

We first examined the concentrations of the complement classical pathway component (C1q), the lectin pathway (MBL) and the alternative pathway (FB) in plasma samples from HBV-ACLF, CHB and control group. The concentrations of C1q was significantly lower in HBV-ACLF group than that in CHB group or in control group (Fig. 1a). The mean concentration of C1q was 50,509 ng/ml in the HBV-ACLF group, but only 114,640 ng/ml in CHB group and 177,001 ng/ml in control group. However, There were no statistical significances of the plasma concentrations of MBL and FB in all groups (Fig. 1b-c), suggesting that the classical pathway plays the more important role in the inflammatory activities of HBV-ACLF. (Table 1).

### The concentrations of complement components C3, C4, their degradation products and sC5b-9 in all groups

Complement C3 and its degradation product C3a were significantly higher in the control group than those in the HBV-ACLF group (Fig. 1d-e). The C3 was 15,653 μg/ml in the control group, 8916 μg/ml in the CHB group and 6568 μg/ml in the HBV-ACLF group. The C4 was 1530 μg/ml in the control group, 1487 μg/ml in the CHB group and 1074 μg/ml in the HBV-ACLF group (Fig. 1f). The C3 and C3a level in ACLF survival group were higher than that in ACLF non-survival group, but without statistical difference. The concentrations of C1q, C3, C3a, C4, C4a and sC5b-9 were significantly higher in the control group than those in the HBV-ACLF group (3.5, 2.4, 2.1, 1.4, 1.3 and 6.0 fold, respectively). (Fig. 1a-h, Table 2).

**Table 2:** Plasma levels of complement components in the HBV-ACLF, CHB group and health control groups

Characteristics	Control group	CHB group	ACLF group	*P* Value
C1q (ng/ml)	177,001 (145157–214,153)	114,640 (34586–198,231)	50,509 (16011–113,313)	< 0.0001
MBL (ng/ml)	3788 (1894–4590)	3785 (2716–4666)	3768 (2402–4749)	0.9571
FB (ng/ml)	236.5 (147.8–303.8)	214.3 (176.0–270.6)	222.4 (141.8–267.4)	0.7615
C3 (μg/ml)	15,653 (12550–16,220)	8916 (3598–16,893)	6568 (1188–15,387)	< 0.0001
C3a (ng/ml)	1755 (1159–1961)	1008 (894–1654)	852 (714–1740)	< 0.0001
C4 (μg/ml)	1530 (498–1843)	1487 (76–2006)	1074 (49–1554)	0.0054
C4a (ng/ml)	2398 (1086–3013)	2304 (1575–3007)	1811 (417–2757)	0.0003
sC5b-9 (ng/ml)	784 (445–2626)	396 (121–2288)	130 (79–1772)	0.0115

Continuous variables were presented as median and the range was shown in brackets

There were no statistical significance of the plasma concentrations of C1q, FB, MBL, C3, C3a, C4, C4a and sC5b-9 between the HBV-ACLF patients with infection and the HBV-ACLF patients without infection. Subgroup analysis showed that the concentrations of C3 was significantly lower in the HBV-ACLF patients with pulmonary infection than that in the HBV-ACLF patients without pulmonary infection (Fig. 1i). There were no statistical significance of the plasma concentrations of C1q, FB, MBL, C3a, C4, C4a and sC5b-9 between the HBV-ACLF patients with pulmonary infection and the HBV-ACLF patients without pulmonary infection.

### The relationship between complement components and clinical parameter

The concentrations of C1q, MBL and FB were not correlated with the prognosis score.

**Fig. 2:**
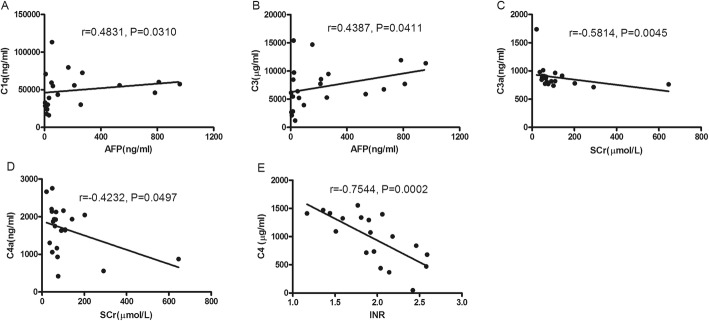
Relationship between complement components and clinical parameter. **a** C1q was observed to be positively correlated with AFP (*P* < 0.05). **b** C3 was observed to be positively correlated with AFP (*P* < 0.05). **c** C3a was observed to be negatively correlated with creatinine (*P* < 0.05). **d** C4a was observed to be negatively correlated with creatinine (*P* < 0.05). **e** C4 was observed to be negatively correlated with INR (*P* < 0.05)

There were no statistical significance of the plasma concentrations of C1q, MBL and FB between the ACLF survival group and ACLF non-survival group. None of them were correlated with ALT, total bilirubin, albumin and HBV DNA. Only the C1q were observed to be positively correlated with AFP (*P* <  0.05). (Fig. 2a).

### The correlation of C3, C4 and their degradation products levels with clinical parameter and prognosis score

C3 was observed to be positively correlated with AFP (*P* <  0.05). C3a was observed to be positively correlated with C3, *r* = 0.4675, but *P* = 0.0504. C3a and C4a were observed to be negatively correlated with creatinine (*P* <  0.05). C4 was observed to be negatively correlated with INR (*P* <  0.05). But none of them were correlated with ALT, total bilirubin, albumin and HBV DNA (Fig. 2b-e).

**Fig. 3:**
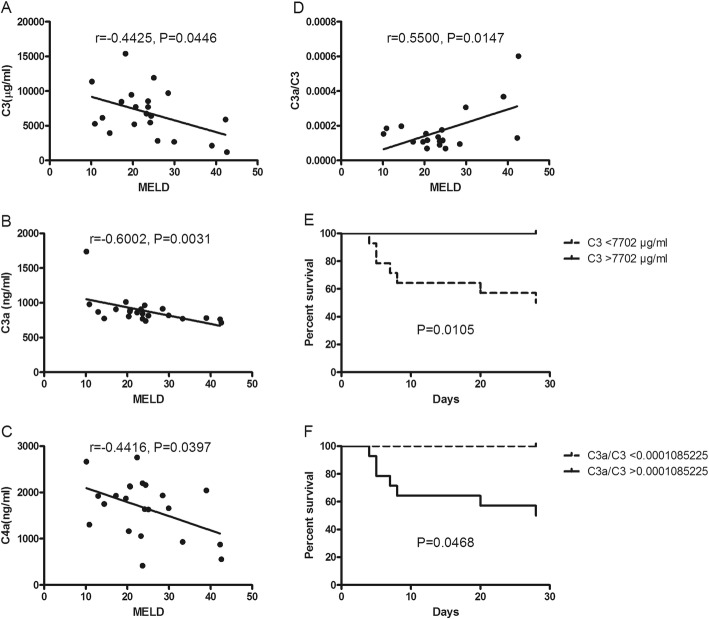
Correlation of C3, C3a and C4a with prognostic scoring systems. **a** C3 was observed to be negatively correlated with MELDs (*P* < 0.05). **b** C3a was observed to be negatively correlated with MELDs (*P* < 0.05). **c** C4a was observed to be negatively correlated with MELDs (*P* < 0.05). **d** The C3a/C3 was positively correlated with MELDs (*P* < 0.05). **e** Kaplan-Meier survival curve was established by the concentrations of C3 (*P* < 0.05). **f** Kaplan-Meier survival curve was established by the concentrations of C3a/C3 (*P* < 0.05)

The concentrations of C3, C3a and C4a were observed to be negatively correlated with MELDs (*P* <  0.05) (Fig. 3a-c). The concentrations of C3, C3a and C4 were observed to be negatively correlated with CLIF-C OFs (*P* <  0.05). The C3a/C3 was positively correlated with MELDs, as well as CLIF-C OFs (*P* <  0.05, Fig. 3d). The optimal cut-point for the concentrations of C3 in predicting death was 7702 μg/ml, with 58.3% sensitivity and 100% specificity (AUROC = 0.764), and for C3a/C3 was 0.0001085 (100% sensitivity and 41.7% specificity, AUROC = 0.736). Kaplan-Meier survival curves classified by the concentrations of C3 = 7702 μg/ml and C3a/C3 = 0.0001085 were compared by the log-rank test (*P* <  0.05, Fig. 3e-f).

### C3 activation in plasma in ACLF group

**Fig. 4:**
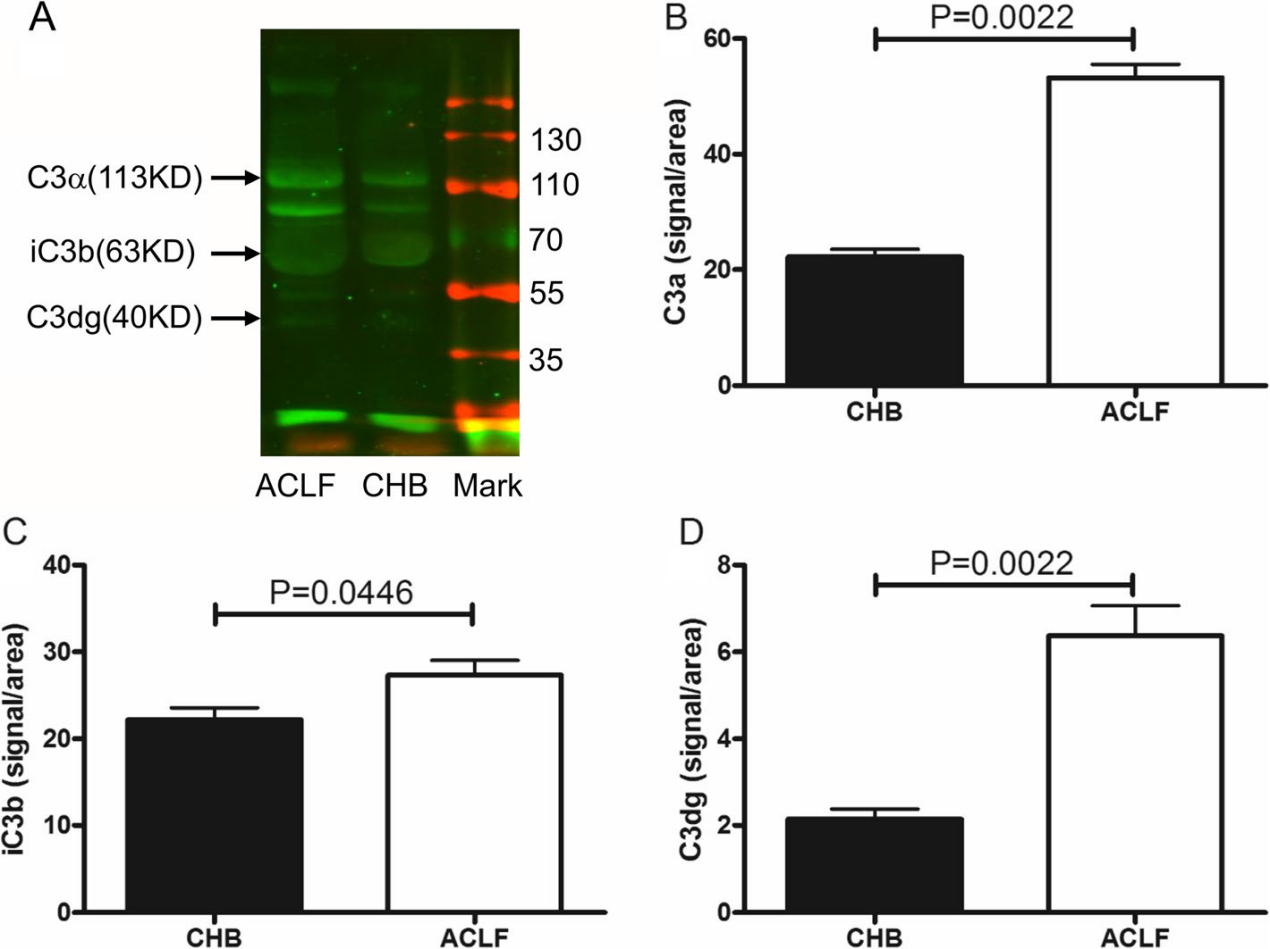
C3 cleavage fragments were analyzed by western blot. **a** The immunoreactive bands corresponding to C3α, iC3b and C3dg are denoted. Under the same C3 concentration, the C3 breakdown products iC3b were more in HBV-ACLF group than the one in CHB group. Prominent iC3b band was found in plasma samples in HBV-ACLF patients. **b** The histogram was showed the difference of the expression of C3a between CHB group and ACLF group (*P* < 0.05). **c** The histogram was showed the difference of the expression of iC3b between CHB group and ACLF group (*P* < 0.05). **d** The histogram was showed the difference of the expression of C3dg between CHB group and ACLF group (*P* < 0.05)

The complement system activation is mediated through the classical, the alternative and the lectin pathways. Each pathway converges at the C3 convertase level resulting in C3 cleavage. To further assess whether or not the C3 activation is involved in ACLF group, the levels of C3 breakdown products in plasma samples were evaluated by Western blot. According to C3 level, we adjusted sample’s volume to ensure the samples’ C3 concentration from two groups was the same for comparison. We repeated western blot test at least three times. Under the same conditions, C3 cleavage fragments iC3b were detected in both groups. Besides, and the level of iC3b was more in ACLF group than the one in CHB group. C3 cleavage fragments iC3b was detected in plasma from both ACLF group and CHB group. Prominent iC3b band was found in plasma samples in ACLF patients. (Fig. 4a-d).

## Discussion

The immune responses and the inflammatory cascades are important pathogenic mechanisms during ACLF. Although most of the plasma complement factors are derived from the liver, the role of the complement system in liver diseases is not completely understood, and few studies were reported for HBV-ACLF before [4].

In response to acute injury, Kupffer cells are activated through complement C3 and C5 receptor signaling pathways [4]. However, there were no reports assessing activation of the complement system in HBV-ACLF patients. In this study, we systematically examined the plasma levels of complement components in HBV-ACLF patients. We firstly showed decreased concentrations of C1q, C3, C3a, C4, C4a and sC5b-9 in HBV-ACLF patients compared to those in the control group. In particular, C3 and C4 levels were significantly lower in HBV-ACLF patients, which is similar to a previous study [15]. Some studies have shown that the MBL level in acute liver failure patients was 40% lower compared with healthy controls, and spontaneous acute liver failure survivors had higher levels of MBL at day 1 and lower levels of L-ficolin by day 3 compared with patients who died or had liver transplant [21]. Despite that, we didn’t find any statistical difference of FB and MBL levels between the HBV-ACLF patients and the control group. This phenomenon may be attributed to the different causes of the disease, but further study is needed clarify the mechanism. The activation of the complement system in response to invading pathogens is initiated through the classical, the alternative and the lectin pathways. The initiators of the three activation pathway are C1q, FB and MBL, respectively. If one pathway is activated, the number of promoter molecules will be significantly reduced compared with the control group because of being consumed. We found that among the three recognition molecules of the complement activation pathways, only C1q, a recognition molecule of the classical pathway activation was significantly reduced in patients with HBV-ACLF, C1q can be produced by activated monocytes/macrophages and immature dendritic cells in addition to liver cells. Therefore, our data indicated that in HBV-ACLF, the classical pathway may play an important role during the disease course.

We investigated the correlation between complement components and prognostic scores in HBV-ACLF patients, and found that C3 and C3a levels were negatively correlated with MELDs and CLIF-C OFs. Therefore, the synergistic protective activity of both C3 and C3a may further improve their diagnostic value in predicting disease progression. The complement components might be a potential biomarker to predict disease outcome in HBV-ACLF patients. Interestingly, the higher level of C3 and C3a means the higher survival rates in HBV-ACLF patients in CLIF-C OF score model, which is a similar to the result of a previous study about C3 level in HBV-ACLF patients [15]. In our previous study, we found that CLIF-C OF is superior to MELDs in predicting HBV-ACLF short-term mortality (AUROC = 0.906 vs AUROC = 0.838, respectively) [18]. In this study, we found that the optimal cut-point for the concentrations of C3 in predicting death was 7702 μg/ml, with 58.3% sensitivity and 100% specificity (AUROC = 0.764), and for C3a/C3 was 0.0001085 (100% sensitivity and 41.7% specificity, AUROC = 0.736). Thus, the plasma levels of C3 and C3a/C3 might be potential novel biomarkers in predicting the outcome of HBV-ACLF.

The complement has been described as a double-edged sword since activation of the complement system significantly contributes to the pathogenesis of various acute and chronic inflammatory diseases, but it also plays a significant role in the resolution of inflammation and tissue repair [22]. C3a has been reported of its anti-inflammatory and regenerative effects for normal liver recovery after toxic injury [23]. Experimental evidence has demonstrated that complement anaphylatoxins C3a and C5a are required for the survival of liver cells during regeneration [24–27]. Further studies to explore the mechanisms responsible for the protective role of C3a during HBV-ACLF were warranted.

We found that C3a might be positively correlated with C3 (*r* = 0.4675, but *P* = 0.0504). It suggests that if HBV-ACLF patients have more functional liver cells, more C3 can be synthesized and more complement effectors C3a can be produced during complement activation.

Hepatorenal syndrome usually indicates poor prognosis of liver failure. The main clinical signs of the hepatorenal syndrome are the progressive oliguria and anuria and the rising levels of the urea nitrogen and blood creatinine. Our study has shown that C3a and C4a were negatively correlated with creatinine. INR is another important biochemical marker, and it is included in MELD and CLIF OF score systems. Our results shown that the levels of C4 were negatively correlated with INR. AFP is not only a marker of hepatocellular carcinoma, but also a biomarker of liver regeneration. We found that C1q and C3 were positively correlated with AFP, which means that the patients with higher C1q and C3 might recover better.

The C3 breakdown products iC3b was more in ACLF group than the one in CHB group, which as observed by Western blot. The increase of iC3b suggests that complement system is more activated in the ACLF group than the CHB group. This may be an important pathogenesis for ACLF. But C3a has been reported its anti-inflammatory and regenerative effects for normal liver recovery after toxic injury. The exact role of complement system in ACLF is unknown. Further study is needed to clarify the mechanism.

## Conclusions

In conclusion, our analysis suggest that the activation of the classical pathway mediated by C1q may play an important role in the pathogenesis of HBV-ACLF. Furthermore, the plasma levels of C3 and C3a may be potential novel biomarkers in predicting the outcome of HBV-ACLF. The role of the complement system activation in host defense and neuro-inflammation in HBV-ACLF patients remains to be fully explored. Highlighting which components of the complement system are important in both protective and inflammatory roles in HBV-ACLF patients could drive development of ACLF-specific immunotherapies.

## Data Availability

Data supporting our findings is contained within the manuscript. Data is available from the corresponding author upon request. Identifying/confidential patient data however will not be shared.

## Abbreviations

ACLF: Acute-on-chronic liver failure
ALT: Alanine aminotransferase
APASL: Asian Pacific Association for the Study of the Liver
AUROC: Area under the receiver operating characteristic curve
CLIF: Chronic liver failure
CLIF-C ACLF: CLIF consortium ACLF score
CLIF-C OF: CLIF consortium organ failure score
CLIF SOFA: CLIF sequential organ failure assessment score
CRP: C reactive protein
C: Complement
FB: Factor B
HBeAg: Hepatitis B virus e antigen
HBV: Hepatitis B virus
HBV-ACLF: HBV-related ACLF
INR: International normalized ratio
IL: Interleukin
MBL: Mannose binding lectin
MELD: The model for end-stage liver disease
TNF-α: Tumor necrosis factor-α
